# Vitamin B Therapy, Methionine Synthase and Cystathionine Beta-Synthase (CBS) Gene Polymorphisms, and Their Impact on Homocysteine and Cardiovascular Events in Ischemic Stroke With Normal Renal Function: A Randomized Controlled Trial

**DOI:** 10.7759/cureus.86727

**Published:** 2025-06-25

**Authors:** Neetu Kataria, Vasantha C Kalyani, Shashi Ranjan Mani Yadav, Mritunjai Singh, Anissa A Mirza, Nitesh Kumar, Niraj Kumar

**Affiliations:** 1 Nursing, All India Institute of Medical Sciences, Rishikesh, Rishikesh, IND; 2 Nursing, All India Institute of Medical Sciences, Deoghar, Deoghar, IND; 3 Biochemistry, All India Institute of Medical Sciences, Rishikesh, Rishikesh, IND; 4 Neurology, All India Institute of Medical Sciences, Rishikesh, Rishikesh, IND

**Keywords:** gene polymorphisms, homocysteine, ischemic stroke, recurrent cardiovascular events, vitamin b supplementations

## Abstract

Background: Hyperhomocysteinemia cases were found to be higher in hilly regions due to factors such as high altitude and dehydration. There is limited research on methionine synthase (MS) and cystathionine beta-synthase (CBS) gene polymorphisms among stroke patients in Southeast Asia. The primary objective of the study was to determine the efficacy of vitamin B therapy in lowering homocysteine levels, and the secondary objective was to investigate the prevalence and impact of MS and CBS gene polymorphisms on treatment outcomes and cardiovascular events.

Methods: A randomized controlled trial was conducted on 90 ischemic stroke patients at a tertiary care hospital, Rishikesh, India. Participants received either vitamin B therapy (B6, B9, B12) or standard therapy for four months. Tools were genetic polymorphisms (polymerase chain reaction-restriction fragment length polymorphism (PCR-RFLP)) testing, National Institutes of Health Stroke Scale (NIHSS), and modified Rankin Scale (mRS) scores, mean homocysteine, mean vitamin B12, and mean folate levels.

Results: The prevalence of MS-AG and CBS-TT polymorphism frequencies was 6% and 12%, respectively. At four months, the vitamin group showed a significant reduction in homocysteine as 8.6 vs. 19 µmol/L in standard therapy, improved mRS scores, and improved vitamin B12 and folate levels with p < 0.001. Vitamin B12 deficiencies and green vegetable intake were key predictors of hyperhomocysteinemia. Clinical outcomes included one recurrent stroke, eight cardiovascular events, and six vascular deaths.

Conclusion: Our observations indicated that vitamin B therapy effectively reduced homocysteine and addressed deficiencies in ischemic stroke patients. However, genetic polymorphism was found to be less prevalent in this hilly region. Combining the role of vitamin B on homocysteine, along with reducing stroke severity and functional disability, leads to early recovery of the stroke patient and reduces rehospitalisation.

## Introduction

Cardiovascular disorders have risen sharply among young South Asians [1], coinciding with an increasing incidence of stroke in India, which ranks fourth in mortality and fifth in disability-adjusted life years (DALYs), accounting for 3.5% of total disabilities [2]. This study emphasizes secondary stroke prevention, focusing on hyperhomocysteinemia (homocysteine levels >15 µmol/L), which is a modifiable and independent risk factor for stroke [3,4]. Hyperhomocysteinemia status is influenced by genetic [5-7], nutritional, renal, and lifestyle factors, which alter the homocysteine metabolic pathway [8].

Insufficient B vitamin intake and genetic mutations (methylenetetrahydrofolate reductase (MTHFR), methionine synthase (MS), cystathionine beta-synthase (CBS)) are key disruptors of homocysteine metabolism. MTHFR and MS genes rely on vitamin B12 and folic acid, while the CBS gene requires vitamin B6 for homocysteine conversion to cystathionine. Of these, MTHFR is a prominent predictor of hyperhomocysteinemia [9]. However, there is limited research on MS and CBS gene polymorphisms among stroke patients in South Asia [6-14]. Studies combining MS and CBS genes are scarce globally, with only three from South and Southeast Asia - one each from India, Pakistan, and Thailand [13-15].

Vitamin B6, B12, and folate levels are inversely associated with homocysteine levels. North Indians are prone to hyperhomocysteinemia due to factors such as high altitude and dehydration [16]. Amid financial constraints in India, vitamin B therapy (B6, B12, and folate) offers a cost-effective solution for high-risk groups prone to cardiovascular disorders and stroke. Recent trials have explored its safety and efficacy in patients with cardiovascular conditions with renal impairment [4,17,18]. However, in India, few online trials have explored the role of only folate supplementation for hyperhomocysteinemia, especially in the Western [13,19] and Northern [6,20] regions.

To the best of our knowledge, no study has been published in India on the effect of vitamin B therapy on homocysteine levels among hyperhomocysteinemic ischemic stroke patients with normal renal function tests. The primary objective was to determine the efficacy of vitamin B therapy in lowering homocysteine levels, and the secondary objective is to investigate the prevalence and impact of MS and CBS gene polymorphisms on treatment outcomes and cardiovascular events with normal renal function, addressing the need for vitamin B interventions on stroke in Sub-Himalayan, hilly region, North India.

This article was previously posted to the Authorea preprint server on 12 December 2022.

## Materials and methods

Study design

This study employed a parallel group randomized controlled design.

Setting

The setting is neurology in the ward and outpatient department and medicine in the patient services of a tertiary care hospital, Rishikesh, Sub-Himalayan hilly region, North India.

Sample

The sample included ischemic stroke patients diagnosed by a neurologist and admitted to the tertiary care hospital in Rishikesh.

Study period

The duration of the study was from September 2020 to September 2021.

Eligibility criteria

Inclusion criteria: Participants included those who were clinically stable ischemic stroke cases within 72 hours of onset, aged 18-70, with hyperhomocysteinemia and normal creatinine levels.

Exclusion criteria: Participants included conditions or medications affecting homocysteine levels, such as migraines, neurodegenerative diseases, specific drugs (e.g., methotrexate, anticonvulsants, lipid-lowering agents), prior vitamin B supplementation, oral contraceptives, or androgens within six months pre-stroke.

Sampling technique and sample size

The sampling technique was a simple random sampling technique. The sample size was 90 calculated for primary objectives by using G*Power (version 3.1; The G*Power Team, Germany) software, with an effect size of 0.83 derived from prior research data (Mean1 = 14.5, Mean2 = 10.7, SD1 = 5.1, SD2 = 4.0) [21], a power of 80%, an alpha of 0.01, and a two-tailed p-value. Each of the two groups in this study enrolled 45 participants.

Interventions

Participants in the vitamin therapy group received an oral tablet containing 5 mg of vitamin B6, 5 mg of vitamin B12, and 500 mcg of folate, administered once daily after a meal, whereas the standard therapy received standard hospital therapy for stroke, following the guidelines established in the HOPE-2 trial, over a four-month duration.

Outcomes

The primary outcome was identified as MS D919G and CBS I278T gene polymorphisms: changes in baseline serum homocysteine levels over four months. Secondary outcomes included assessing the recurrence of major cardiovascular events, vascular deaths, the change in serum vitamin B12 and folate levels, and modified Rankin scale (mRS) scores from baseline to four months. 

Randomization

**Figure 1:**
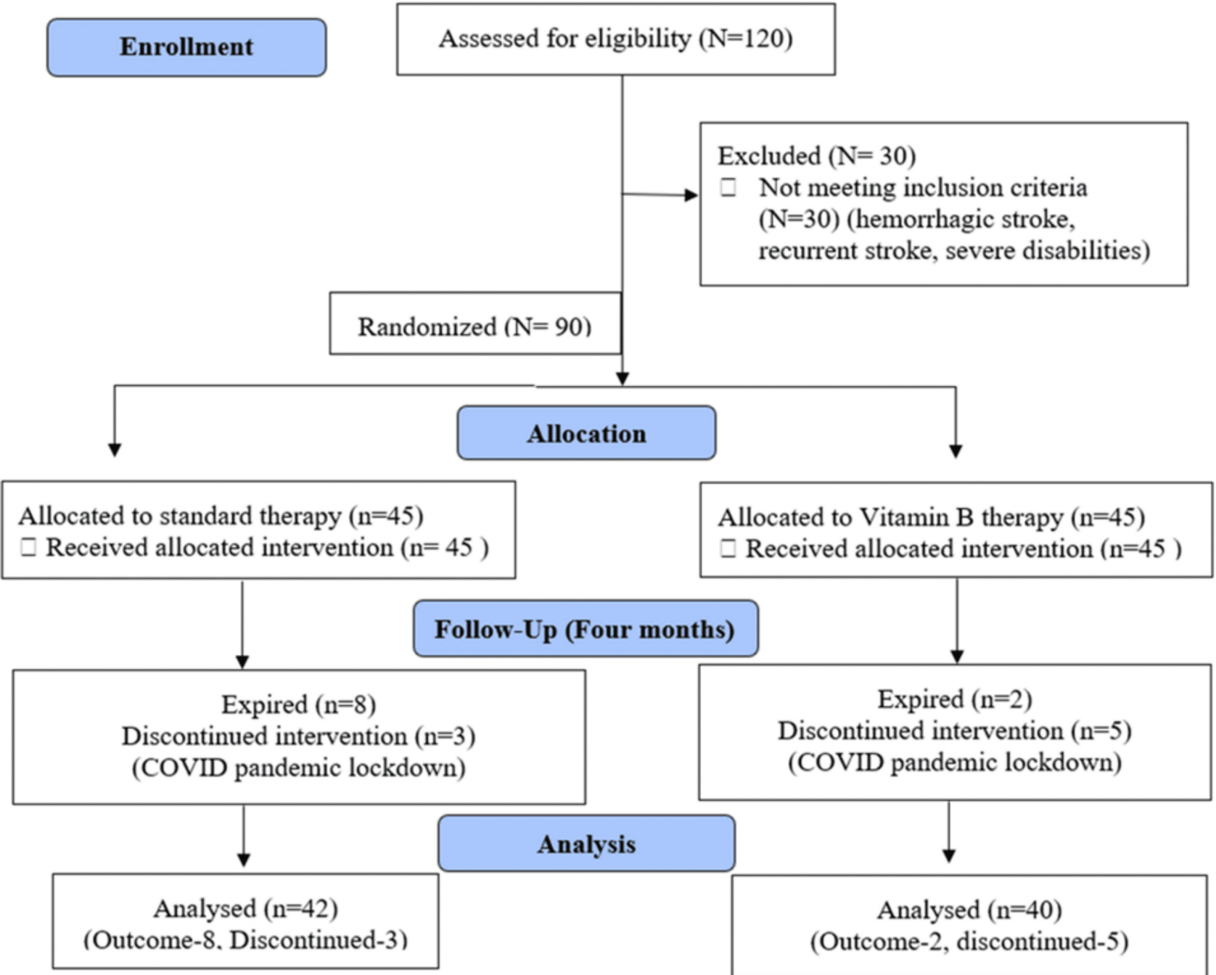
CONSORT flow chart. CONSORT: CONsolidated Standards Of Reporting Trials

Sequence generation: The study adhered to the CONSORT (CONsolidated Standards Of Reporting Trials) 2010 guidelines for its design and reporting [22] (Figure 1). Participants were recruited from the emergency department of a tertiary care hospital, Rishikesh. Randomization was conducted using a computer-generated random number table, created through the website (sealed.envelope.com), with SEED number 59486749384304. A colleague, who was not listed as an author, generated the random number list.

Allocation concealment: No allocation concealment was used in the study as no sealed envelopes were used to allocate particular groups by using an open random number table method. Ninety participants were randomly allocated into two groups in a 1:1 ratio by an independent allocator-nursing officer, with 45 participants in the vitamin B therapy and 45 in standard therapy.

Blinding: The treating physician and the statistician remained blinded to the randomization process and interventions to ensure unbiased outcomes as data were not entered in terms of group A and group B; hence, the statistician did not know about treatment within groups. However, blinding of participants was not possible due to the study design.

Ethical approval

The ethical approval for this trial has been obtained from an independent institutional ethical committee (IEC) (no. AIIMS/IEC/19/1135) at a tertiary care hospital and the Clinical Trials Registry - India (CTRI) (no. CTRI/2020/03/024293). Ethical guidelines were followed in good clinical practices, the Declaration of Helsinki, and the Indian Council of Medical Research (ICMR) in this study. Written informed consent was obtained from all participants before enrollment. Confidentiality and anonymity of participant data were strictly maintained throughout the study.

Statistical methods

The software Statistical Product and Service Solutions (SPSS, version 23; IBM SPSS Statistics for Windows, Armonk, NY) was used for data analysis and appropriate descriptive and inferential statistics. The normality of the data was assessed through Kolmogorov-Smirnov testing, with a p-value > 0.05 considered as normally distributed. The data were normalized for the standard therapy genetic polymorphism by following the Hardy-Weinberg equilibrium (HWE) (by using the wpcalc.com website). Further, intention-to-treat analysis was performed for the study participants.

Participants flowchart

A total of 90 participants were randomly assigned to both vitamin and standard therapy. Thus, the sample used after dropout cases (N = 82) had 40 participants in the vitamin therapy group, and 42 in the standard therapy group were analyzed, with an attrition rate of 8.8%, although it was found homogenous for both groups with a p-value of 0.45. Missing data accounted for 8.8% of the total dataset, distributed across both groups. Since the missingness appeared to be listwise deletion/multiple imputation was applied to minimize bias, the results were found to be consistent between imputed and non-imputed analyses.

Recruitment/data collection and laboratory techniques

Participants were recruited between September 2020 and September 2021 from neurology and medicine inpatient services at a tertiary care hospital located in the sub-Himalayan region of North India.

Data collection began with a subject datasheet and clinical variables obtained through self-reported methods. Stroke-related scores, including NIHSS, mRS, and GCS, were recorded for all participants. All assessment tools used were standardized and freely available in the public domain. Validation of these tools for the study setting was confirmed by five experts, including two neurologists and three nursing departments. The reliability of the NIHSS and mRS tools was measured at 0.92 and 0.94, respectively, in this setup.

Baseline investigations included serum homocysteine (µmol/L), folate (ng/mL), and vitamin B12 (pmol/L) levels, analyzed using a chemiluminescent immunoassay on the Advia-Centaur XP immunoassay system. Genetic polymorphism screening for all 82 participants was conducted using the polymerase chain reaction-restriction fragment length polymorphism (PCR-RFLP) technique [23].

Researcher studied the MS D919 gene (211bp), which includes three genotypes - AA (homozygous wild), AG (heterozygous mutant), and GG (homozygous mutant), whereas the CBS I278T gene (282bp) has four genotypes - TT (homozygous wild), TC (heterozygous wild), TC (heterozygous mutant), and CC (homozygous mutant). Details of the genetic testing protocol were mentioned in supplementary material attached (Appendix).

## Results

Baseline data

We included 82 participants in the analysis at baseline and at four-month follow-ups. Table 1 shows that all socio-demographic and clinical variables at baseline were matched between both groups of stroke participants with a p-value > 0.05.

**Table 1:** Baseline distribution of socio-demographic and clinical characteristics (N = 82). & - chi-square test, Mann-Whitney U test, @ - Student's 't' test, DM - diabetes mellitus, NIHSS - National Institute of Health Stroke scale, GCS - Glasgow Coma Scale, * p value < 0.05 considered as significant.

S. No.	Variables	Category	f (%)		p value
			Vitamin group (n=40)	Standard therapy (n=42)	
1	Age	Mean±SD	49.1±14.2	51.1±14.7	0.5^@^
2	Gender	Male	30 (75.5%)	27 (64.4%)	0.2
3	Habitat	Rural	29 (73.3%)	28 (66.6%)	0.4
4	Site of stroke	Left hemisphere	21 (53.3%)	18 (44.4%)	0.1
5	Hypertensive	(>140/90 mmHg)	26 (66.6%)	23 (55.5%)	0.2
6	Type-II DM	Present	8 (20%)	12 (28.8%)	0.3
7	Type of tobacco abuse	Smoking	18 (44.4%)	16 (37.7%)	0.6
8	Green leafy vegetables (times per week)	Mean±SD	3.2±1.2	3.6±1.4	0.1^@^
9	Baseline NIHSS score (Total-42)	Mean±SD	12.4±6.0	15.1±5.4	0.06^&^
10	Baseline GCS score (Total-15)	Mean±SD	11.8±2.9	10.8±3.2	0.1^@^
11	Days of hospital stay	Mean±SD	13.0±10.4	18.0±12.7	0.07^&^
12	Creatinine (mg/dL)	Mean±SD	1.0±1.8	0.75±0.2	0.8^&^
13	Total cholesterol (mg/dL)	Mean±SD	164.9±53.2	146.1±38.8	0.6^@^

Effect of vitamin B therapy on homocysteine levels

Table 2 indicates that the mean homocysteine levels reduced significantly at four months in the vitamin group (8.6 ± 3.7 µmol/L) as compared to the standard therapy among ischemic stroke patients (19.0 ± 11.1µmol/L), with p-value < 0.01* (95% CI: -13.8; -6.9).

**Table 2:** Distribution of outcome variables at baseline and four months (N = 82). $ - Student's 't' test and Mann-Whitney test value, & - Fisher exact test, tHcy - total homocysteine levels, mRS - modified Rankin scale, CVD - cardiovascular disorders, * p value < 0.05 considered as significant

S. No.	Variable	Vitamin group (n=40) (Mean± SD)	Standard therapy (n=42) (Mean±SD)	‘t’ value (p value)	95% CI (lower; upper)
1	Homocysteine (tHcy) levels (Baseline) (µmol/L)	23.8±9.3	25.2±11.8	-0.72 (0.4)	(-6.0; 2.8)
	At 4 months	8.6±3.7	19.0±11.1	-5.9 (<0.001*)	(-13.8; -6.9)
2	Vitamin B 12 levels (Baseline) (pmol/L)	586.8±559.4	676.4±622.2	-0.5 (0.5)^$^	(-337.7; 158.2)
	At 4 months	713.4±415.2	585.8±608.4	-2.6 (<0.01*)^$^	(-91.0; 345.8)
3	Folate (B9) levels (Baseline) (ng/mL)	7.8±4.3	6.4±2.8	1.8 (0.07)	(-0.1; 2.9)
	At 4 months	8.9±2.5	6.1±2.0	5.7 (<0.01*)	(1.8; 3.7)
4	mRS score (Baseline) (Total score: 6)	3.8±1.9	4.4±1.1	-1.9 (0.06)	(-1.3; 0.00)
	At 4 months	1.1±1.1	2.3±1.5	-3.8 (<0.01*)^$^	(-1.7; -0.6)

Hence, vitamin B therapy was found to be effective in reducing homocysteine levels; in terms of percentage, these levels were 64% in the vitamin therapy group vs 26% in the standard therapy group from baseline to four months of duration.

Polymorphism of MS D919G and CBS I278T in homocysteine metabolizing enzymes

Table 3 shows the frequency distribution of genotypes of both MS D919G and CBS I278T in both groups of ischemic stroke patients found under the HWE (p-value = 0.09). The percentage of the genotypes of MS D919 AG-heterozygous was 2.5% vs 9.5% among the vitamin therapy vs standard therapy, respectively, whereas the CBS TC-heterozygous genotype was 15% vs 9.5% among the vitamin therapy vs standard therapy, respectively. Analysis was done by using a chi-square test, and no significant association was found at p-values of 0.3 and 0.7, respectively.

**Table 3:** Frequency distribution of both genotypes of methionine synthase (MS) and cystathionine beta-synthase (CBS) genes (N = 82). *p value < 0.05 considered as significant, & - Fisher's exact test

S. No.	Gene polymorphisms	Vitamin group (n=40) f (%)	Standard therapy (n=42) f (%)	p value	HWE - p value
1	MS-D919G, Heterozygous mutant-AG	1 (2.5%)	4 (9.5%)	0.3^&^	0.09
2	CBS-I278T, Heterozygous wild-TC	6 (15%)	4 (9.5%)	0.7^&^	0.09

Hence, both groups were matched in genotypic distribution for both genes involved in the homocysteine metabolism pathway for this study’s participants.

Effect of vitamin B therapy on vitamin B12 levels and folate levels

Table 2 shows that vitamin B therapy led to a significant mean improvement in vitamin B12 levels and folate levels from baseline to four months in the vitamin group, as compared to the standard therapy at p-value < 0.01.

Hence, the affordable and safe vitamin B therapy caused a significant improvement in the vitamin B12 and folate levels in the vitamin group compared with the standard therapy from baseline to four months among ischemic stroke patients.

Effect of vitamin B therapy on mRS scores (mRS for functional disability)

Table 2 illustrates that vitamin B therapy contributed to a significant mean reduction in mRS scores (functional disability) from baseline to four months in the vitamin group at p-value < 0.01, respectively. Therefore, it can be concluded that vitamin B therapy was effective in reducing mRS scores, accelerating patient recovery, and improving the patient’s condition in the vitamin group.

**Figure 2:**
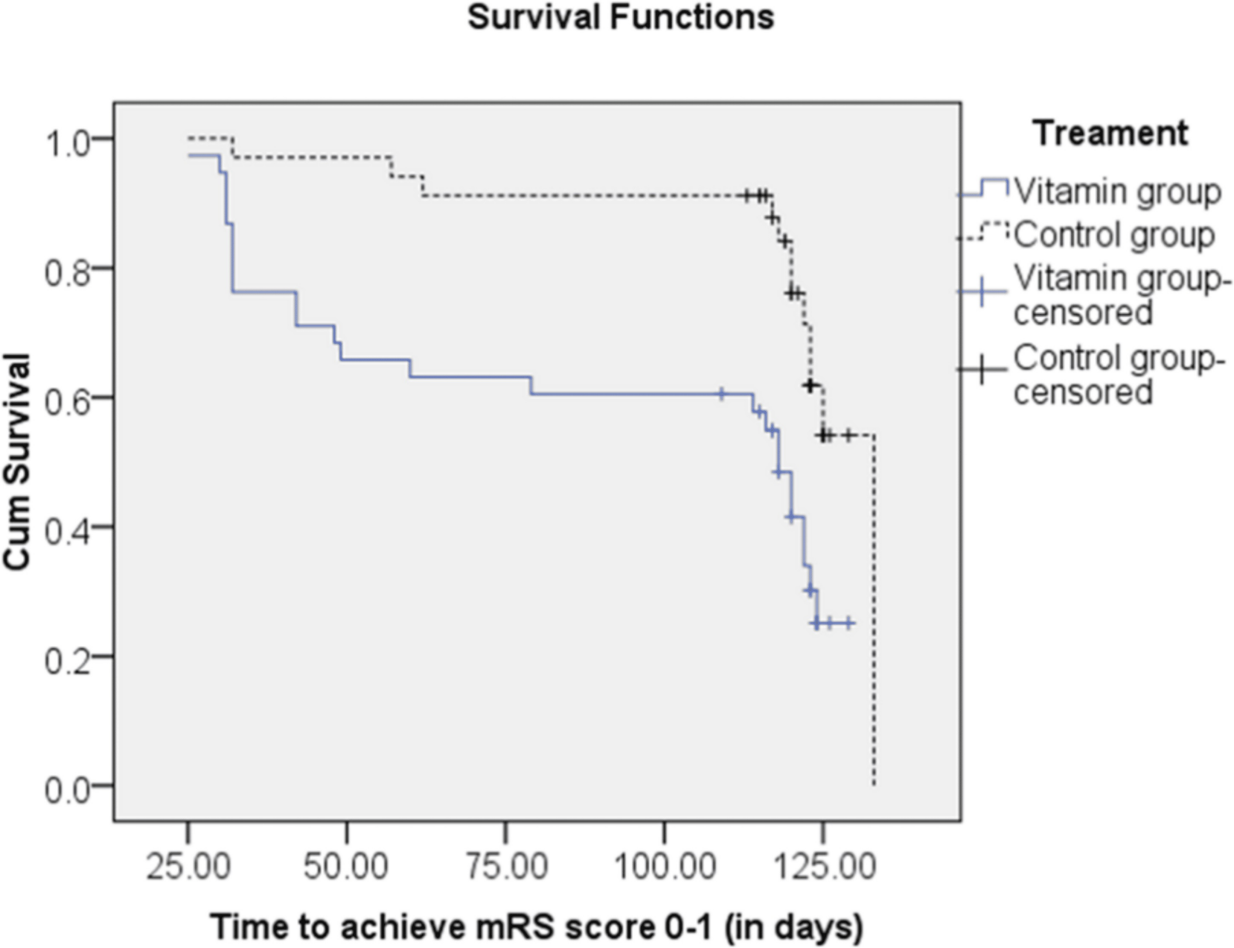
Kaplan-Meier curve for achieving a modified Rankin scale (mRs) score of 0-1 (in days). Equality for achieving an mRS score of 0-1 (in days) curves for the ischemic stroke patients between both the groups are shown in the Kaplan-Meier curve and were tested with log-rank test, * p value < 0.05.

Figure 2 represents the Kaplan-Meier estimates of the probability of achieving an mRS score of 0-1 (in days) among the vitamin group vs. standard therapy, respectively. At 120 days (four months), the hazard ratio of not being able to achieve an mRS score of 0-1 for the vitamin group, as compared with the standard therapy, as HR = 0.37 (95% CI: 0.168; 0.849, p = 0.009*) by using the log-rank test, with p = 0.001*. It can be interpreted from the above curve that the vitamin group was 63% more likely to achieve an mRS score of 0-1 earlier than the standard therapy, respectively.

Effect of vitamin B therapy on the recurrence of stroke, CVD events, and vascular death

In Table 4, out of the 82 participants, only one (2.4%) recurrent stroke event was reported, specifically for a participant in standard therapy. A total of eight recurrent cardiovascular events occurred, three (7.5%) in the vitamin group and five (11.9%) in the standard therapy; however, the results were not found to be significantly different (p value = 0.7).

**Table 4:** Frequency distribution of cardiovascular events (N = 90). & - Fisher's exact test, CVD - cardiovascular disorders, * p value < 0.05 considered as significant.

S. No.	Cardiovascular events	Vitamin group (n=45) f (%)	Standard therapy (n=45) f (%)	p value
1	Recurrence of stroke (1)	0 (0%)	1 (2.4%)	0.9^&^
2	Recurrence of CVD events (8)	3 (7.5%)	5 (11.9%)	0.7^&^
3	In-hospital vascular death (6)	1 (2.5%)	5 (11.9%)	0.3^&^
4	Death due to any other origin (4)	1 (2.5%)	3 (7.1%)	0.61^&^

A total of six in-hospital vascular death events occurred, one (2.5%) in the vitamin therapy group and five (11.9%) in the standard therapy; however, the results were not found to be significantly different (p-value = 0.31).

The total number of deaths due to other reasons, including sepsis and septic shock, was three (7.1%) in the standard therapy and one (2.5%) in the experimental group (due to septic shock). However, the results were not found to be significantly different (p-value = 0.61). From the above-mentioned findings, it can be interpreted that the distribution of the recurrent major CVD events and deaths did not differ between the two groups, with a p-value > 0.05. However, the recurrent event numbers were lower for the vitamin group as compared with the standard therapy, respectively.

Polymorphisms’ interaction with mean tHcy levels, vitamin B12 levels, and folate levels at baseline and four months

Table 5 illustrates that, in the vitamin group (N = 40), the interaction of the MS D919G genotypes with mean homocysteine levels, vitamin B12 levels, and folate levels at baseline (N = 82) and at four months (N = 40) was found to be non-significant with a p-value 0.52, 0.31, and 0.89, respectively, for interaction without any adjustment to the covariates (with adjustment data mentioned in regression analysis interpretation). Similarly, in the vitamin group, the interaction of the genotypes of the CBS I278T with mean homocysteine levels, vitamin B 12 levels, and folate levels at baseline (N = 82) and at four months (N = 40) was also found non-significant at a p-value of 0.53, 0.83, and 0.70, respectively, for interaction. Additionally, the MS and CBS genotypes did not modify the efficacy of vitamin B therapy on homocysteine, vitamin B12, and folate levels mentioned in the present trial.

**Table 5:** Polymorphisms interaction with mean tHcy levels, vitamin B12, and folate levels at baseline and four months (N = 82). & - Student's ‘t’ test and Mann-Whitney U test, tHcy - total homocysteine levels, * p value considered as significant ≤ 0.05 (mean difference calculated as follows: mean at four months - mean at baseline), p value for interaction means - mean difference in two genotypes (MS or CBS) among the vitamin group.

At baseline		At four months			
(N=82) Mean±SD		Vitamin group (n=40)		Standard therapy (n=42)	
		Mean±SD	Mean difference	Mean±SD	Mean difference
Baseline tHcy (µmol/L)					
MS D919G gene					
AA (n=69)	24.06±8.35	9.47±2.11	13.31±6.63	19.09±8.18	5.09±1.81
AG (n=3)	18.90±0.96	9.20	9.03	28.00	5.20
p value	p=0.292	p=0.89	p value for interaction=0.52	p=0.29	p=0.95
CBS I278T gene					
TT (n=64)	23.57±7.97	9.68±2.14	13.46±6.83	20.00±8.31	5.35±1.67
TC (n=8)	26.09±10.47	8.02±0.68	11.45±4.81	12.63±0.47	4.42±2.57
p value	p=0.410	p=0.96	p value for interaction=0.53	p=0.14	p=0.38
Baseline vitamin B12 (pmol/L)					
MS D919G gene					
AA (n=69)	548.32±505.92	696.57±334.4	334.2±218.04	489.8±440.05	211.70±170.06
AG (n=3)	168.10±100.01	1500	500	310	100
p value	p=0.12^&^	p=0.10^&^	p value for interaction=0.31^&^	p=0.88^&^	p=0.82^&^
CBS I278T gene					
TT (n=64)	613.3±598.8	717.9±370	348.9±250.60	492.8±329.0	224.35±126.8
TC (n=8)	453.3±292.5	716.0±258	270.5±131.06	400±247.5	440.00±308.4
p value	p=0.52^&^	p=0.99^&^	p value for interaction=0.83^&^	p=0.73^&^	p=0.13^&^
Baseline Folate (ng/mL)					
MS D919G gene					
AA (n=69)	6.82±3.39	8.92±2.36	2.31±1.71	6.01±1.43	1.24±1.04
AG (n=3)	9.52±2.29	9.20	2.28	7.46	0.97
p value	p=0.17	p=0.91	p value for interaction=0.89^&^	p=0.33	p=0.76^&^
CBS I278T gene					
TT (n=64)	6.90±3.42	8.99±2.35	2.29±1.73	6.04±1.42	1.29±1.65
TC (n=8)	7.12±3.27	8.96±2.43	2.45±1.51	6.23±1.86	0.60±0.43
p value	p=0.82	p=0.98	p value for interaction=0.70^&^	p=0.83	p=0.13^&^

Multiple linear regression analysis for predicting variables for total homocysteine levels

Table 6 shows the impact of all clinico-genetic variables and blood parameters on the total homocysteine levels. By adjusting covariates and keeping them constant (the choice of covariates was taken from previous literature) [6], the results of linear regression analysis showed that the consumption of green leafy vegetables (OR = 0.80) and baseline blood vitamin B12 levels (OR = -0.25) significantly determined the homocysteine levels in the present dataset. The adjusted R2 (corrected goodness-of-fit (model accuracy)) for the model was 0.17, signifying that 17% of the deviation in the homocysteine levels was clarified by these two variables.

**Table 6:** Multivariate linear regression analysis performed with homocysteine as the dependent variable, keeping all other predictive factors constant (N = 82). * p value considered as significant < 0.05, adjusted R2 value - 0.17

S. N.	Variable	Unstandardized B	Beta	‘t’	p value	95% CI (lower; upper)
1	Green leafy vegetables consumption	2.02	0.80	2.51	0.01*	(0.42; 3.61)
2	Baseline vitamin B12 levels	-0.005	-0.25	-2.30	0.02*	(-0.009; 0.000)
3	Baseline folate levels	0.294	0.10	0.953	0.34	(-0.320; 0.909)
4	Alcohol abuse	4.53	0.20	196	0.054	(-0.052; 9.11)
5	MS D919G gene polymorphism	-6.80	-0.14	-1.39	0.1	(-16.51; 2.90)
6	CBS I278T gene polymorphism	-2.02	-0.06	-0.58	0.5	(-8.90; 4.85)

## Discussion

The aim of the study was to assess the impact of vitamin B therapy, MS and CBS gene polymorphisms on homocysteine levels, and cardiovascular events in ischemic stroke patients. The results of the study revealed that mean homocysteine levels significantly reduced in the vitamin therapy group (8.6 vs 19 µmol/L) compared to the standard therapy group at four months among ischemic stroke patients. The frequency distribution of the MS D919 AG-heterozygous gene polymorphism was 2.5% vs 9.5% among the vitamin therapy vs standard therapy, respectively, whereas the CBS TC-heterozygous genotype was 15% vs 9.5%, respectively. Similarly, vitamin B supplementations significantly improved mean vitamin B12 and folate levels in the vitamin therapy group compared to the standard therapy group.

Magnitude of gene polymorphisms

Hyperhomocysteinemia is a major risk factor for vascular occlusive diseases and CVDs in India [20]. Sub-Himalayan vegetarians are particularly vulnerable [6,7,16]. The present study found that the MS D919G AG genotype polymorphism at 6%, much lower than in Pakistan (23%) [14] and West India (44%) [13]. Similarly, for the CBS I278T TC genotype, findings were 12% [14], aligning with findings from Pakistan (9.6%) and Turkey (11%) [24], but absent in West India [13]. These variations may be due to ethnic diversity, historical migrations, and differences in genetic testing techniques [13-15,24].

Association of polymorphism and homocysteine levels

No significant association was found between the MS D919G and CBS I278T polymorphisms and homocysteine levels, consistent with prior studies [6-10]. The minimal polymorphism level detected could be due to genetic and environmental differences [6,7,11,13-15,25]. Additionally, inflammation and atherosclerosis may contribute to CVD risk beyond homocysteine levels [7,16].

Impact of vitamin B therapy on homocysteine levels

Vitamin B therapy group significantly reduced homocysteine levels by 15.2 ± 10.5 µmol/L over four months, exceeding reductions in the VISP (2 µmol/L) [26] and VITATOPS (-3.18 µmol/L) [17] trials. It also improved vitamin B12 and folate levels, supporting prior findings from the HOPE-2 trial [27,28]. The therapy’s efficacy may be due to dosage, shorter treatment duration, and the absence of folic acid fortification.

Impact of vitamin B therapy on vitamin B12 and folate levels

The administration of vitamin B therapy yielded significant improvements in vitamin B12 levels and folate levels among the vitamin group of ischemic stroke patients. These findings align with previous studies, including the HOPE-2 trials [27], and corroborate earlier research that demonstrated substantial enhancements in vitamin B12 and folate levels.

Effect on stroke, CVD events, and vascular death

Only one stroke occurred in the standard therapy, while prior trials reported higher rates (16.8%-21%). CVD events were 7.5% in the vitamin therapy group vs 11.9% in controls, higher than in previous trials (2.5%-2.6%). Vascular deaths were 2.2% in the therapy group and 11.11% in controls, aligning with prior studies. The COVID-19 pandemic likely influenced these outcomes as other comorbidities and nutritional deficiencies lead to higher case fatality rates [18,27,29].

Harms: If any adverse events happen due to such intervention, they must be reported to the IEC and the trial stopped. However, in the current study, no harm was reported.

Limitations of the study

This trial was conducted upon a small but calculated sample and a single-centered study that limits its generalizability to other healthcare settings. Due to COVID-19 constraints (lockdown), only specific samples were collected, and blinding was not feasible, which potentially introduces performance bias, particularly in subjective outcomes such as self-reported adherence or functional assessments. Findings suggest dietary habits influence vitamin B12 and folate levels, declaring further research. The low prevalence of MS and CBS gene variants limits the ability to draw firm conclusions about their role. The frequencies of MS (6%) and CBS (12%) polymorphisms were low, which limits the statistical power (type II error) to detect gene-treatment interactions. In this study, the confounding variable of unadjusted baseline NIHSS imbalance (p=0.06) may bias mRS results. Adherence to vitamin supplementation was assessed by collecting empty medication packets. This self-reported measure is prone to bias and is not sufficiently robust for a clinical trial. The duration of the four-month follow-up was relatively short for assessing cardiovascular events and stroke recurrence. Longer-term outcomes were needed for generalizability. The trial was also conducted during the COVID-19 pandemic, which may have influenced patient characteristics and outcomes. Although green leafy vegetable intake was measured, broader dietary patterns, alcohol intake, socioeconomic status, and comorbidities were not comprehensively assessed or controlled for, which may confound the relationship between vitamin therapy and homocysteine levels.

Strengths of the study

The present study was a randomized controlled trial, which is a gold standard in clinical research. This unique trial of this region ensured strong adherence to vitamin B therapy, resulting in a significant reduction in homocysteine and other outcomes with no harmful side effects, minimal withdrawals, and maintained statistical power, which further enhances generalizability and replicability of the study methods into different settings.

## Conclusions

This unique study demonstrates that oral vitamin B supplementation significantly reduces homocysteine levels, improves functional disability, and enhances vitamin B12 and folate levels within four months among hyperhomocysteinemia and ischemic stroke patients with normal kidney function, without any adverse effects. These findings support vitamin B safety and efficacy as part of post-stroke rehabilitation in hilly regions, North India. The study also suggests that ethnic and geographical factors may influence hyperhomocysteinemia prevalence, independent of economic or dietary differences. The prevalence of genetic polymorphism was lower in this hilly region. Notably, the effects of vitamin B therapy on homocysteine were unaffected by MS and CBS genotype polymorphisms. These insights underscore the potential of vitamin B supplementation in clinical practice and highlight the need for larger trials and more genetic studies in hilly regions.

## Appendices

Genetic testing protocol

The major steps included in the genetic testing protocol are as follows: (1) DNA extraction via Qiagen kit, DNA quantification, and gel electrophoresis; (2) PCR technique than gel electrophoresis and imaging in the UV doc machine; (3) RFLP technique than gel electrophoresis and imaging in the UV doc machine; and (4) final three images on gel electrophoresis and imaging in the UV doc machine

DNA extraction protocol from the Qiagen kit

Step 1: Blood sample kept outside freezer at room temperature, and 200 µL of blood taken into the Eppendorf tube, and mix 20 µL of proteinase K enzyme into it. Hence, the total amount becomes 220µL in the Eppendorf tube.

Step 2: 200 µLof AL buffer was mixed and vortexed. The sample was incubated at 56 degrees for 10 minutes in the incubator.

Step 3: 200 µL ethanol (96-100%) was added and vortexed into the same Eppendorf tube.

Step 4: Lysis was transferred into the mini-column and centrifuged at 8,000 RPM for one minute, and then the collection tube was discarded.

Step 5: Attach a new collection tube, 500 µL of AW1 buffer was added and centrifuged at 8,000 RPM for one minute, and the collection tube was discarded.

Step 6: Attach a new collection tube, 500 µL of AW2 buffer was added and centrifuged at 14,000 RPM for three minutes, the spin column was removed and transferred into the 1.5 mL Eppendorf tube.

Step 7: 200 µL of AE buffer was added and then kept incubated for one minute at room temperature. Then, it was centrifuged at 8,000 RPM for 1-2 minutes, removed its upper spin column, and stored at -20 degrees Celsius.

Step 8: If you would like to increase the amount of DNA sample, repeat Step 7.

DNA quantification was performed by the machine: 

Name of equipment: Microplate multimode reader

Model/make:

Bioscreen:

Date of installation: 2.03.2015

Number of samples: 16

(Gel-electrophoresis machine)

Name of equipment: Vertical gel electrophoresis

Model/make: M/S Vitan Medical

Date of installation: 05.06.2015

Number of samples: Large comb - 15, small comb - 8

Gel-UV doc machine:

Name of equipment: Chemiluminescence & Gel imaging & analysis system

Model/make: Bio Digital Pvt. Ltd.

Date of installation: 2.09.2013

Polymerase chain reaction (PCR) machine:

Name of equipment:

Master cycler nexus gradient

Model/make:

Eppendorf

Date of installation: 08.07.2017

Number of samples: 96

1) MS D919G gene (211 bp)

The patient’s 2 mL venous blood sample was stored at -20 degrees for DNA extraction by using the Qiagen kit.

DNA image capturing process: 4 µL of properly mixed DNA sample was taken, then 1 µL of Bromophenol tracking dye was added, and 5 µL was loaded into the 1% gel. The gel was made with 0.4 mg agarose in a 40 mL TAE buffer solution (with 4 µL Ethidium Bromide (EtBr) stain) and melt it into the microwave. After cooling down at room temperature for 20 minutes to set, pour the gel into the tray. Gel electrophoresis runs at 90 mV for 45 minutes, and the image is captured in a UV Gel Doc machine.

Protocol for gel run for PCR after DNA extraction

Primers for the MS Gene

Gene: Primers

MS D919G Fw: “(Fw-5’-TGT TCC CAG CTG TTA GAT GAA AAT C-3’)”

MS D919G Rw: “(Rw-5’- GAT CCA AAG CCT TTT ACA CTC CTC-3’)”

Protocol for a master mix for conducting PCR after DNA extraction

PCR mix protocol

Master Mix (MM)

10 x 1 = 10 µL

Forward primer (FP)

1 x 1 = 1 µL

Reverse primer (RP)

1 x 1 = 1 µL

Nuclease-free water (NFW)

6 x 1 = 6 µL

Total solution = 18 µL + 2 µL of sample DNA = 20 µL

Protocol of the MS gene (PCR run temperature conditions)

Steps

Step 1: 94 degrees for three minutes

Step 2: 45 cycles

Denaturation at 94 degrees for 30 seconds

Primer annealing at 58 degrees for 30 seconds

Primer extension at 72 degrees for 30 seconds

Step 3: Final primer extension at 72 degrees for 5 minutes

Hold for 10 degrees

Gel electrophoresis for PCR and capturing image process: 5 µL of PCR product was loaded with premixed dye into the master mix in a gel. 1.5% gel made up of 0.6 mg agarose in 40 mL TAE buffer solution at 90 mV for 50 50-minute run, and capture image into the UV doc machine.

Protocol of the master mix for conducting the RFLP technique for the MS gene with enzyme: Hae-II (same as the above MTHFR gene RFLP)

Total solution: 10 µL

Its incubation at 37 degrees was 14-16 hours.

Gel electrophoresis for the RFLP-MS gene and final image capturing process: 8 µL was loaded into a 1.5% gel. This gel is made up of 0.6 mg agarose in a 40 mL TAE buffer solution and then run at 80 mV for 50 minutes, and the image was captured under a UV Gel Doc machine.

2) CBS A1298T gene (282bp)

The patient’s 2 mL venous blood sample was stored at -20 degrees for DNA extraction by using the Qiagen kit.

DNA image capturing process: 4 µL of properly mixed DNA sample was taken, 1 µL of Bromophenol tracking dye was added, and 5 µL was loaded into the 1% gel. The gel was made with 0.4 mg agarose in a 40 mL TAE buffer solution (with 4 µL Ethidium Bromide (EtBr) stain) and melted in the microwave. After cooling down at room temperature for 20 minutes to set the gel, pour the gel into the tray. Gel electrophoresis runs at 90 mV for 45 minutes, and the image was captured in a UV Gel Doc machine.

Protocol for gel run for PCR after DNA extraction

Primers for the CBS gene

Gene: Primers

CBS I278T Fw: “(Fw -5’ATA GAA TAT CGA GGC ATG TCC AGG CG 3V;)”

CBS I278T Rw: “(Rw: 5’TGG GGC CCA GGG TCA GCC AGG CTC C 3’)”

Protocol for a master mix for conducting PCR after DNA extraction

PCR Mix Protocol

Master Mix (MM)

10 x 1 = 10 µL

Forward primer (FP)

1 x 1 = 1 µL

Reverse primer (RP)

1 x 1 = 1 µL

Nuclease-free water (NFW)

6 x 1 = 6 µL

Total solution = 18 µL + 2 µL of sample DNA = 20 µL

Protocol of CBS gene (PCR run temperature conditions) 

Steps

Step 1: 94 degrees for three minutes

Step 2: 30 cycles

Denaturation at 94 degrees for 30 seconds

Primer annealing at 72 degrees for 30 seconds

Primer extension at 72 degrees for 1 min

Step 3: 72 degrees for 10 minutes

Hold on 10 degrees

Gel electrophoresis for PCR, and capturing image process: 5 µL PCR product loaded with premixed dye into the master mix in the gel. The 1.5% gel is made up of 0.6 mg agarose in 40 mL TAE buffer solution and run at 90 mV for 50 minutes, and the image was captured under the gel electrophoresis machine.

Protocol of master mix for conducting RFLP technique for CBS gene with enzyme: -BSr1 (same as above mentioned for MHTFR RFLP technique)

Total solution = 10 µL

It is incubated at 37 degrees overnight for 14-16 hours.

Gel electrophoresis for the RFLP-CBS gene and final image capturing process: 8 µL was loaded into the 1.5% gel. This gel is made up of 0.6 mg agarose in a 40 mL TAE buffer solution and run at 80 mV for 50 minutes, and the image was captured under a UV Gel Doc machine.

The result showed the genotypic distribution of these two genes as follows: MS D919G and CBS I2798T

Homozygous wild-AA; Homozygous wild-TT; Heterozygous Mutant-AG; Heterozygous wild-TC; Homozygous mutant-GG; Heterozygous mutant-TC; Homozygous mutant-CC
